# **α**-RgIB: A Novel Antagonist Peptide of Neuronal Acetylcholine Receptor Isolated from *Conus regius* Venom

**DOI:** 10.1155/2013/543028

**Published:** 2013-02-27

**Authors:** Maria Cristina Vianna Braga, Arthur Andrade Nery, Henning Ulrich, Katsuhiro Konno, Juliana Mozer Sciani, Daniel Carvalho Pimenta

**Affiliations:** ^1^CAT/CEPID, Instituto Butantan, Avenida Vital Brasil 1500, 05503-900 São Paulo, SP, Brazil; ^2^Ministério da Ciência, Tecnologia e Inovação, Esplanada dos Ministérios, Bloco E, 70067-900 Brasília, DF, Brazil; ^3^Departamento de Bioquímica, Instituto de Química, Universidade de São Paulo, Av. Lineu Prestes 748, 05508-900 São Paulo, SP, Brazil; ^4^Institute of Natural Medicine, University of Toyama, 2630 Sugitani, Toyama 930-0194, Japan; ^5^Laboratório de Bioquímica e Biofísica, Instituto Butantan, Avenida Vital Brasil 1500, 05503-900 São Paulo, SP, Brazil

## Abstract

*Conus* venoms are rich sources of biologically active peptides that act specifically on ionic channels and metabotropic receptors present at the neuromuscular junction, efficiently paralyzing the prey. Each species of *Conus* may have 50 to 200 uncharacterized bioactive peptides with pharmacological interest. *Conus regius* is a vermivorous species that inhabits Northeastern Brazilian tropical waters. In this work, we characterized one peptide with activity on neuronal acetylcholine receptor (nAChR). Crude venom was purified by reverse-phase HPLC and selected fractions were screened and sequenced by mass spectrometry, MALDI-ToF, and ESI-Q-ToF, respectively. A new peptide was identified, bearing two disulfide bridges. The novel 2,701 Da peptide belongs to the cysteine framework I, corresponding to the cysteine pattern CC-C-C. The biological activity of the purified peptide was tested by intracranial injection in mice, and it was observed that high concentrations induced hyperactivity in the animals, whereas lower doses caused breathing difficulty. The activity of this peptide was assayed in patch-clamp experiments, on nAChR-rich cells, in whole-cell configuration. The peptide blocked slow rise-time neuronal receptors, probably **α**3**β**4 and/or **α**3**β**4**α**5 subtype. According to the nomenclature, the new peptide was designated as **α**-RgIB.

## 1. Introduction

Marine mollusks from *Conus* genus may produce from 50 up to 200 biologically active molecules that can be injected in the prey to capture or be employed as defense and/or escape mechanisms to deter competitors. The peptide toxins, called conopeptides, are composed of 10–40 amino acids (including nonnatural amino acids) and are abundant in the venom. Peptides presenting a rigid structure due to more than one disulfide bridges are common, being called conotoxins. These peptides act specifically on ionic channels and/or neuromuscular receptors [1, 2]. 

Conotoxins are classified according to three schemes: the similarities between the endoplasmatic reticulum signal sequence of the conotoxin precursors (gene superfamilies), the cysteine patterns of conotoxin mature peptide regions (cysteine frameworks), and the specificities to pharmacological targets (pharmacological families) [3, 4].

Conopeptides of the pharmacological family *α*, which acts on neuronal acetylcholine receptor, have been found in the A, D, L, M, and S gene superfamilies [5, 6]. 

Typically, *α*-conotoxins are peptides with 12 to 16 amino acid residues and two disulfide bridges, presenting the pattern CC-C-C. These peptides are competitive antagonists of the nicotinic acetylcholine receptors (nAChR) and display high selectivity by subtypes of this receptor [5, 7–9]. After the blockage of the muscular acetylcholine receptor, the *α*-conotoxins significantly decrease the amplitude of the motor end plate postsynaptic potentials in vertebrates, paralyzing the prey [10].

In the Brazilian tropical coast, there are approximately 18 species of cone snails [11]. *Conus regius* (Gmelin, 1791) is a vermivorous species that inhabits rock and coral deep waters of Florida (USA), Central America, and the Northeast and East coast of Brazil, including Fernando de Noronha archipelago [12].

In this work we described a novel peptide from *Conus regius* venom, belonging to the *α*-conotoxins family. This peptide blocks the neuronal acetylcholine receptors on PC12 cells, which comprise *α*3*β*4 and/or *α*3*β*4*α*5 subtypes receptors, probably target of the peptide. 

## 2. Material and Methods

### 2.1. Reagents

All the employed reagents were of analytical grade and were purchased from Sigma Co (St Louis, MO, USA), unless otherwise stated. 

### 2.2. Animals and Venom

Specimens of *C. regius* were collected at Fernando de Noronha Archipelago, Pernambuco, Brazil. The Brazilian Environmental Agency (IBAMA—Instituto Brasileiro do Meio Ambiente e dos Recursos Naturais Renováveis) license numbers were 030/2000 and 087/2001, and the process number was 02001, 000775/00-00. Venom was extracted from the specimens as previously described [13]. The crude venom was obtained by dissection of the venom duct gland and then freeze-dried and stored at −80°C. 

Voucher material is deposited in the malacological collection of Zoology Museum of University of São Paulo, São Paulo, Brazil.

### 2.3. Peptide Fractionation and Purification

A reversed-phase binary HPLC system (LC-8A, Shimadzu Co., Japan) was used for sample fractionation. The lyophilized crude venom powder was solubilized into 0.1% trifluoroacetic acid (TFA) and aliquots were loaded in a Shim-pack Prep-ODS C18 column (Shimadzu, 3 *μ*m, C18, 300 Å, 250 × 20 mm) in a two-solvent system: (A) trifluoroacetic acid (TFA)/H_2_O (1 : 1000) and (B) TFA/Acetonitrile (ACN)/H_2_O (1 : 900 : 100). The sample was eluted at a constant flow rate of 8 mL·min^−1^ with a 0 to 60% gradient of solvent B over 60 min. The HPLC column eluates were monitored by a Shimadzu SPD-10A detector scanning 220 nm. 

For *α*-RgIB purification, the interest peak was fractionated in a Merck C18 column (300 × 4.6 mm), in a 19 to 21% B gradient over 20 min, at a constant flow rate of 8 mL·min^−1^. A subsequent purification step was still necessary to obtain the peptide. This purification was conducted in a Merck C18 column (300 × 4.6 mm), in an isocratic elution at 35% B (TFA/methanol/H_2_O 1 : 900 : 100) at a constant flow rate of 1 mL·min^−1^.

### 2.4. Mass Spectrometry Analysis

Molecular mass analyses of the peaks and the peptides were performed on a micro-LC-MS Ettan (Amersham Biosciences, Sweden) coupled in a Q-ToF Ultima API (Micromass, Manchester, UK) and/or by MALDI-TOF mass spectrometry on a Ettan MALDI-ToF/Pro System (Amersham Biosciences, Sweden).

The analysis in the micro-LC-MS Ettan (Amersham Biosciences, Sweden) was performed in a *μ*RPC C2/C18 ST 1.0/150 column (Amersham Biosciences, Sweden), with two solvents: (A) formic acid (FA)/H_2_O (1 : 1000) and (B) FA/ACN/H_2_O (1 : 900 : 100). The sample was eluted at a constant flow rate of 50 *μ*L·min^−1^ with a 5 to 65% gradient of solvent B over 60 min. Q-Tof operated under positive ionization mode. For MALDI-TOF analyses, a-cyano-4-hydroxycinnamic acid was used as matrix. 

### 2.5. “De Novo” Peptide Sequencing

Mass spectrometric “de novo” peptide sequencing was carried out in positive ionization mode on a Q-TOF Ultima API fitted with an electrospray ion source (Micromass, Manchester, UK). Briefly, the amounts of previously lyophilized peptide were dissolved in 50 mM ammonium acetate, reduced with 50 mM DTT, alkylated by 150 mM iodoacetamide, and hydrolyzed by 25 nM trypsin, according to slight modifications of Westermeier and Naven [14]. The reaction products were then lyophilized and dissolved in 50% ACN, containing 0.1% FA and injected into the source at 5 *μ*L·min^−1^ by a Hamilton infusion pump, or directly injected using a Rheodyne 7010 sample loop coupled to a LC-10A VP Shimadzu pump operating at 20 *μ*L·min^−1^ constant flow rate. The instrument control and data acquisition were conducted by MassLynx 4.0 data system (Micromass, Manchester, UK) and experiments were performed by scanning a mass-to-charge ratio (*m*/*z*) of 50–1800 using a scan time of 2 s applied during the whole chromatographic process. The mass spectra corresponding to each signal from the total ion current (TIC) chromatogram were averaged, allowing an accurate molecular mass determination. External calibration of the mass scale was performed with NaI. For the MS/MS analysis, collision energy ranged from 18 to 45 and the precursor ions were selected under a 1-*m/z* window. 

### 2.6. Biological Activity

The biological activity of *α*-RgIB was determined in Swiss Webster mice (5.5 to 7 g body weight) by observation of the behavioral disorders after intracranial injection [15] of the peptide diluted in NaCl 0.9%, in concentration of 0.1, 0.5 and 1 nmol. Alterations were compared to animals injected with NaCl 0.9% (control). All animals were observed by 60 min. 

### 2.7. Patch Clamp

BC_3_H1 cells, mouse myocytes which express nicotinic acetylcholine receptors, were acquired by ATCC (CRL-1443) and maintained in culture according to Sine and Taylor [16] to electrophysiological experiments. In order to verify the subtype of neuronal nicotinic receptor that the peptide acts, PC12 cells were employed and maintained in culture according to Greene et al. [17].

Individual cells were subjected to a patch-clamp, at a whole cell configuration, according to Hamill et al. [18] and Urlich et al. [19]. Cells were maintained in an extracellular solution containing 25 mM HEPES, 5.3 mM KCl, 144.8 mM NaCl, 1.2 mM MgCl_2_, 2.38 mM CaCl_2_, and 10 mM glucose (pH 7.4). A recording electrode was filled with intracellular solution containing 25 mM HEPES, 141 mM KCl, 10 mM NaCl, 2 mM MgCl_2,_ and 1 mM EGTA (pH 7.4). Experiments were carried out at room temperature (20–24°C).

Throughout the experiment, the membrane potential was clamped at a −60 mV for BC_3_H_1_ cells and −70 mV for PC12 cells, holding potential using an Axon Axopatch amplifier (Molecular Devices, California, USA). Data were recorded and digitized by Clampex 8.2 software (Molecular Devices, California, USA) and plots were made using Origin 7.0 software (OriginLab Corp., Northampton, MA). 

Control currents were performed with 1.5 mM carbamylcholine, using the cell-flow technique [20]. After the carbamylcholine administration, the peptide (10 *μ*M) was incubated on cells, and then another dose of the agonist was incubated [21]. 

### 2.8. Data Fitting, Statistical Analyses, and Sequence Alignment

When data fitting was performed, results were presented as the calculated value ± standard deviation (SD). Otherwise, data correspond to the mean of three individual experiments. Peptide sequence alignment was performed using ClustalW software [22]. The 3D model of the peptide *α*-RgIB, as well as three PDB deposited 3D solution structures, was created by I-TASSER [23, 24].

## 3. Results

### 3.1. Purification

**Figure 1:**
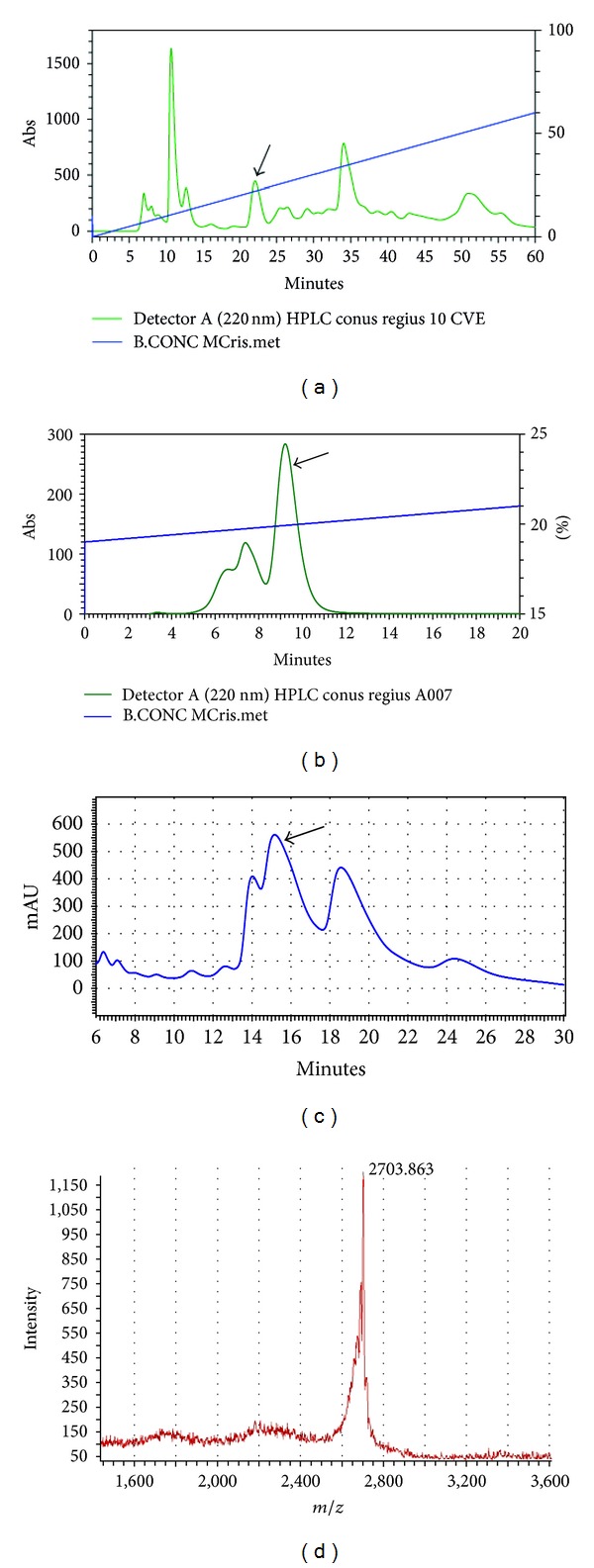
(a) Representative RP-HPLC of the crude *C. regius* venom. The arrow indicates the peak of interest. (b) Representative RP-HPLC of the selected peak (arrow), indicating the presence of impurities. (c) Isocratic elution of the isolated the selected fraction from chromatogram B. The arrow indicates the peak of interest. (d) MALDI-TOF/MS profile of the purified peptide.

The crude venom from *C. regius* was fractionated by RP-HPLC, as shown in Figure 1(a). Some peaks could be detected along the profile, and the arrow indicates the peak of interest. Two subsequent chromatographic steps were necessary to purify the peptide (Figures 1(b) and 1(c)), under the conditions described material and methods section. After the third step of purification, the purity and the molecular mass of the peptide were assessed by MALDI-TOF/MS (Figure 1(d)).

### 3.2. “De Novo” Peptide Sequencing

**Figure 2:**
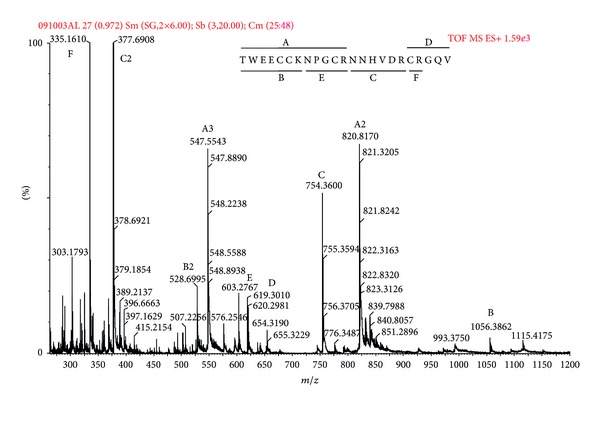
Representative ESI-Q-TOF/MS profile of the trypsin digested purified peptide. The deduced sequence is printed above the spectrum, together with the tryptic peptides (A–F). The MS profile indicates the tryptic peptides and the charge states.

**Figure 3:**
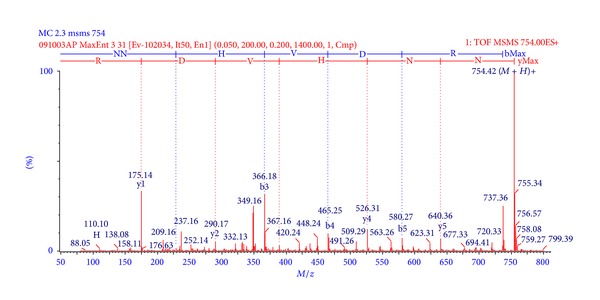
MaxEnt3 deconvoluted annotated representative MS/MS profile of the CID spectrum of tryptic peptide C, from Figure 2. y and b series are annotated above the spectrum, as well as other fragments.

After cysteine bridge reduction and alkylation, the reaction product was digested with trypsin. The obtained peptides were submitted to MS/MS analyses (Figure 2) and ions were selected and fragmented by collision with argon (CIF), yielding daughter ion spectra (Figure 3) that was processed with BioLynx and manually checked for accuracy of interpretation. Since the digestion allowed peptides with missed cleavage sites, it was possible to assemble the fragments without the aid of another digestion with a different enzyme. The sequenced peptides, their charge states, and theoretical molecular mass are presented in Table 1. 

The peptide sequence was determined to be TWEECCKNPGCRNNHVDRCRGQV. This sequence has 4 cysteine residues with pattern CC-C-C, typical from conotoxins of framework I [25]. This peptide was named *α*-RgIB, according to the guidelines for conotoxins nomenclature ConoServer and has been assigned the following UNIPROT accession number: C0HJA8 [3, 6, 26]. 

### 3.3. Sequence Features

**Figure 4:**
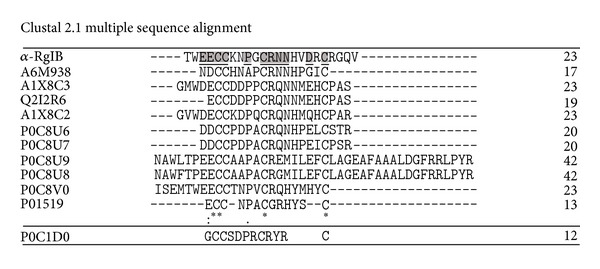
ClustalW alignment of *α*-RgIB and the closest phylogenetic *α*-conotoxins relatives (calculated according to supplemental Figure 3).(*) Consensus; (:) highly conserved; (.) conserved. The bold underlined amino acid residues of *α*-RgIB were also considered to be conserved. A6M938: *α*-conotoxin-like Lp1.10 C. *leopardus*/homology; A1X8C3: *α*-conotoxin-like Lp1.7 C. *leopardus*/transcript; Q2I2R6: *α*-conotoxin-like Lt1.3 C. *litteratus*/transcript; A1X8C2: *α*-conotoxin-like Lp1.8 C. *leopardus*/transcript; P0C8U6: *α*-conotoxin-like PuSG1.1 C. pulicarius/transcript; P0C8U9: *α*-conotoxin-like Pu1.5 C. *pulicarius*/transcript; P0C8U8: *α*-conotoxin-like Pu1.4 C. *pulicarius*/transcript; P0C8U7/*α*-conotoxin-like PuSG1.2 C. *pulicarius*/trasncritpt; P0C8V0: *α*-conotoxin-like Pu1.6 C. *pulicarius*/transcript; P01519: *α*-conotoxin GIA C. *geographus*/protein; P0C1D0: *α*-conotoxin RgIA, C. *regius*/protein. (Key: UniProt Accesion code: toxin/Conus species/evidence level).

**Figure 5:**
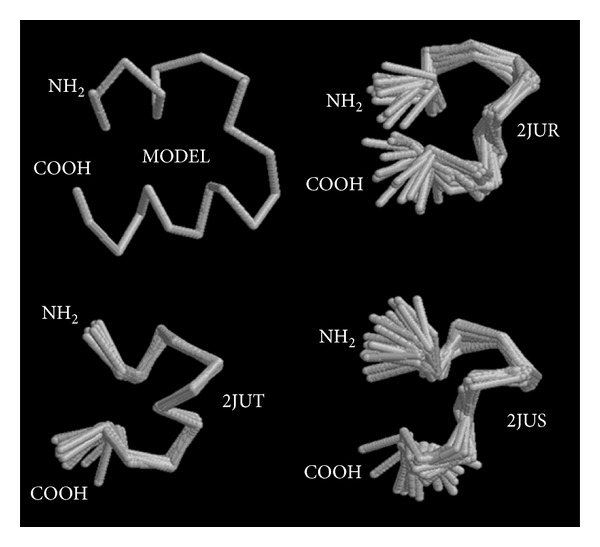
I-TASSER model of *α*-RgIB and PDB NMR superimposed structures of *α*-RgI-A.

A sequence alignment was performed with all *α*-conotoxins available at UniProt (supplementary Table 1 of the supplementary material available online at http://dx.doi.org/10.1155/2013/543028). Based on this large alignment, a phylogeny was constructed (supplementary Figure 1) and the peptide sequences present at the branch containing *α*-RgIB were realigned (Figure 4). ClustalW standard annotations consider, as expected, the Cys residues as consensus (*), and the Glu at the 8th aligned position, as being highly conserved (:). Moreover, according to the algorithm standard notation, the Pro residue at the 13th aligned position is also conserved (.). Among the UNIPROT database, the SwissModel tool could not identify any suitable template for structure prediction of *α*-RgIB, therefore an external application was used.  Figure 5 presents a 3D model of *α*-RgIB; created by I-TASSER [23, 24], as well as three PDB deposited 3D solution structures of *α*-RgIA (P0C1D0) mutants, a conotoxin that specifically and potently blocks the *α*9*α*10 nAChR [27]. In spite of *α*-RgIB N- and C-terminal extensions and longer interbridge peptide sequence, the model and the structures are tridimensionally related, for example, a C-shaped structure, held by the Cys-bridges.

### 3.4. Biological Activity

The *in vivo* biological activity of the peptide was assessed by means of intracranial injection in Swiss Webster mice. Following 1 nmol injection, the animals displayed a hyperactive behavior, defecating and urinating all the time, which was not observed for the control group that received saline solution. Auditory stimuli, for example, a hand-clap or hitting the cage, also triggered the hyperactive behavior. Interestingly, the lower doses (0.1 and 0.5 nmol), caused the animals to have difficulty in breathing. Although the peptide promoted behavioral disorders, it was not lethal to the animals.

### 3.5. Patch Clamp

Whole-cell voltage clamp measurement was used to verify the ion currents on acetylcholine receptors. BC_3_H1 cells, which express the acetylcholine muscle type receptors on the surface, and PC12, which terminally differentiate in neurons and express nicotinic neuronal receptors [21] were selected for the experiments. Carbamylcholine, a stable and well-characterized analogue of acetylcholine, was used as an agonist [28], for it elicits a fast activating current that rapidly desensitizes during the application. 

10 *μ*M *α*-RgIB was not able to induce any change in the ion currents on BC_3_H_1_ cells (data not shown), as well as a higher dose (30 *μ*M) of the peptide. d-tubocurarine (a classic nicotinic receptor antagonist) was used as a positive control and successfully to block this channel (data not shown).

**Figure 6:**
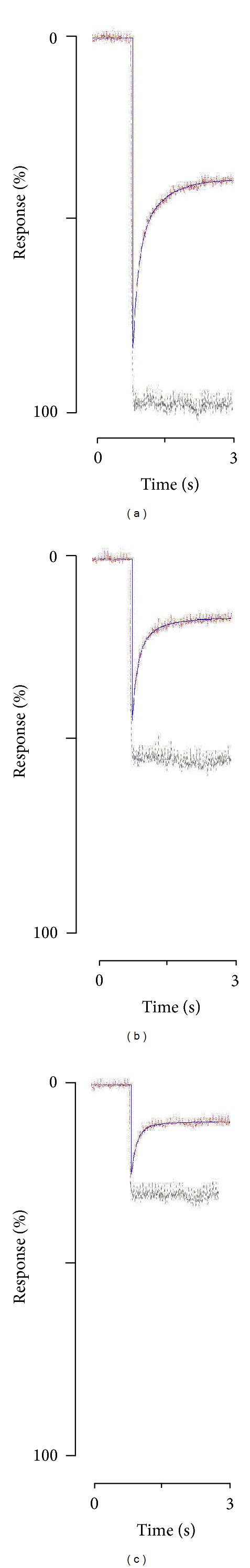
PC12 whole-cell characteristic patch clamp currents (expressed as a percentage of response to 1.5 mM carbamoylcholine (CBC)) (a), 1.5 mM CBC + 10 *μ*M *α*-RgIB (b), and 1.5 mM CBC (c). Cells were kept at −70 mV.

After the incubation of the peptide with PC12 cells, fast and slow desensitization of the receptor was observed.  Figure 6(b) shows that on neuronal slow rise-time receptors, *α*-RgIB is able to block the ion current by 40%, compared to cells stimulated with carbamylcholine (Figure 6(a)). The blockage was irreversible and persistent, once the current does not recover after a new application of the agonist, carbamylcholine (Figure 6(c)).

## 4. Discussion

Conotoxins are classified according to the similarities between the signal sequence of the conotoxin precursors (gene superfamilies), the cysteine patterns of conotoxin mature peptide regions (cysteine frameworks), and the specificities to pharmacological targets [3, 4].

This new peptide was termed *α*-Rg-IB because the peptide acts on neuronal acetylcholine receptors (“*α*”), was extracted from a *Conus regius* specimen (“Rg”), displays a cysteine framework I—CC-C-C (“I”), and was the second peptide discovered with both being from *C. regius* with a cysteine framework I (“B”) [25]. 

*α*-RgIA was the first *α*-conotoxin described from *C. regius*, acting on neuronal nicotinic receptors. This peptide has been thoroughly characterized in terms of its primary and three-dimensional structures [29], as well as regarding its biological effect, for example, the blockage of the *α*9*α*10 nAChR [30, 31]. *α*-RgIA and *α*-RgIB come from the same animal, belong to the same toxin family, and possess similar biological effects; however, their amino acid sequences differ. Figure 4 shows the ClustalW alignment of *α*-RgIB and its closest phylogenetic relatives (supplementary material), besides *α*-RgIA, which was not considered to be similar (according to MEGA5), but was manually inserted in the figure for the benefit of sequence comparison. *α*-RgIA is shorter, both in the N- and C-terminal flanking regions, as well as in the inter-Cys-bridge region. Nevertheless, in a considerably small universe of possibilities (8 out 12, since 4 amino acids are necessarily Cys), *α*-RgIA and *α*-RgIB bare considerable similarities: the Pro, at the 13th aligned position, and the charged residues at the 17th and 18th aligned positions. It is noteworthy to mention that, in spite of the phylogenetic analyses, all conotoxins listed in Figure 4 (except *α*-RgIA and *α*-RgIB) come from other *Conus* species: *C. leopardus *(A6 M938, A1X8C2, A1X8C3), *C. litteratus* (Q2I2R6), *C. pulicarius *(P0C8U6, P0C8U7, P0C8U8, P0C8U9, and P0C8V0) and *C. geographus* (P01519). Moreover, only P01519 has been detected at the protein level and has been characterized as active on the muscular nicotinic receptors [32]. 

Besides *α*-RgIA, the following toxins have been isolated from *C. regius*: P85009; P85010; P85011; P85012; and P85013, all *α*-conotoxin-like peptides belonging to superfamily A; P85016; P85017; P85018; P85019; P85020; P85021 and P85022, all belonging to the M-superfamily of conotoxins [33]. Moreover, our group has also identified two conotoxins, as well: Rg11a, belonging to the I_1_ superfamily (P84197, [34]); and Rg9.1, belonging to the P-superfamily (Q8I6V7; direct submission).

There is no high level of homology between *α*-RgIB and the conopeptides described until the present moment; therefore, the identification of a proper 3D structure to serve as a template for homology modeling is deprecated. Instead, a structure was predicted by using I-TASSER server [23, 24].  Figure 5 shows that, in spite of the low homology with *α*-RgIA, *α*-RgIB model assumed the same basic shape as the NMR determined structures of the *α*-RgIA mutants, available at the PDB database [27]. 

Regarding the rather unique amino acid sequence of *α*-RgIB and thoroughly analyzing our data, we could not rule out the possibility that one of the glutamic acid (Glu) residues of this novel conotoxin would be a gamma-carboxyglutamic acid residue (Gla). Our suspicions arouse from the slightly higher deviation between the theoretical and calculated molecular mass values for A and B ions (Table 1), that could reflect that a side chain carboxylation and not an N-terminal acetylation would be present. Moreover, conotoxins are known for presenting posttranslation modifications, Gla included [35–38] and, even though the MALDI data of the crude peptide support the proposed peptide sequence, MALDI ionization is also a source of facile decarboxylation for Gla residues [39]. Our future experiments with *C. regius* conotoxins (*α*-RgIB included) will clarify this matter.

*α*-conotoxins bind to nicotinic acetylcholine receptors. The subgroup *α*3/5 of *α*-conotoxins, from piscivorous *Conus*, has the motif CCX_3_CX_5_C and can cause paralysis of the prey by the binding on muscle nicotinic receptors. Another subgroup, *α*4/3, that present the motif CCX_4_CX_3_C, bind on neuronal nicotinic receptors. The main subgroup of *α*-conotoxins is *α*4/7, with motif CCX_4_CX_7_C. These peptides bind in all classes of nicotinic receptors: muscular (e.g., *α*-conotoxin EI), homomeric neuronal (e.g., *α*-conotoxins PnIB), and heteromeric neuronal (*α*-conotoxins MII and AuIB) [9].

Neuronal nicotinic acetylcholine receptors (nAChRs) belong to the pentameric superfamily of Cys-loop ligand gated ionic channels. They are composed of either homomeric *α* or heteromeric *α* and *β* subunits assembled from a family of 12 distinct neuronal nicotinic subunits (*α*2–*α*10; *β*2–*β*4) [5]. The combination of subunits *α*2, *α*3, and *α*4 with *β*2 and *β*4 results in a functional receptor, as well *α*7, *α*8, and *α*9 homomeric receptors [40, 41]. In our experiments, it was verified by RT-PCR (supplemental Figure 3) that the pool of PC12 cells expressed *α*3, *α*5, *α*7, *β*2, and *β*4 subunits of neuronal nicotinic receptors, the same pattern found by Sargent [40] in PC12 cells. However, in spite of *α*-Rg-IB affinity by the PC12 nicotinic receptors, there are still other neuronal nicotinic receptors that may be higher affinity targets for these toxins that were not explored in the present work. 

AuIB, from *Conus aulicus*, which is also an *α*-conotoxin, blocks the *α*3*β*4 receptors; however, the currents can be recovered after the toxin washing [42]. In our experiments, the no-recovery of *α*-RgIB is probably due to the irreversible action of the peptide on the receptor. Successive applications of the agonist (carbamolycholine), in control experiments, did not cause recovery of the ion currents on slow rise-time receptors (data not shown). Besides the irreversible action, the peptide may also be able to prolong the desensitization time of the receptor, since the repeated CBC administration on *α*-RgIB-treated PC12 cells was not able to recover the initial current, which is either caused by the irreversible binding of a low affinity toxin or the prolonging of the desensitization time of the receptor (or both).

Sudweeks and Yakel [43] showed that *α*3, *α*7, and *β*2 subunits of nAChR are correlated to fast rise-time receptors. The slow desensitization is a characteristic of *α*3*β*4 receptor, while *α*3*β*2 receptor is from fast desensitization [44]. The fast desensitization receptors, on PC12 cells, contain *α*3*β*2, *α*3*β*2*α*5, and *α*7 subunits, while slow desensitization receptors are formed by subunits *α*3*β*4 and *α*3*β*4*α*5. *α*-RgIB was able to inhibit the currents elicited by carbamolycholine on PC12 cells, mainly on the slow desensitization component, which comprise, in our model, *α*3*β*4 and *α*3*β*4*α*5 receptors. 

The intracranial injection assay was performed to investigate whether there would be any direct activity of the toxin in the central nervous system (CNS), once peptides can promote behavioral alterations by acting on receptors and ionic channels on CNS. These alterations can indicate activities on specific ionic channels. For example, *ω*-conotoxin GVIA causes trembling on the mice, which indicates an action on calcium ionic channels [45]. The *α*-nicotinic acetylcholine receptor (nAChR) is associated to attention-deficit/hyperactivity disorder [46] which corroborates our observations of *α*-RgIB-treated hyperactive mice.

In conclusion, we have isolated a novel conotoxin from *Conus regius* and, by means of a combination of biochemical, structural and pharmacological assays were able to classify this peptide in the *α*-family and named it *α*-RgIB. There are still several peptides to be explored in the *C. regius* venom, as our previous qualitative investigations have shown [34] and the current study has focused on the biochemical characterization of one such novel peptide. Further studies are still necessary to better characterize the structural and pharmacological properties of *α*-RgIB.

## Supplementary Material

The accompanying figures and tables provide information that subside the results presented in the main text. These data consist of one CLUSTALW alignment of all deposited **α**-conotoxins, one explanatory table with the toxin description and organism of origin and the resulting molecular phylogenetic tree built (by MEGA) on the alignment data. This tree branches provide a better visualization of the possible phylogenetic relations among the toxins. This tree served to locate the branch in which **α**-RgIB sits. The closest phylogenetic relatives were than reanalyzed for homology and presented in the main text. Moreover, this material also presents the expression profile of the nicotinic receptors detected in the employed cell cultures, which is important for proper pharmacological characterization.

## Figures and Tables

**Table 1:** Theoretical and experimental *m/z* values for the tryptic peptides obtained after the enzymatic digestion of *α*-RgIB.

Ions	[M + H]^+^		[M + 2H]^2+^		[M + 3H]^3+^		Sequence
	Exp.	Theor.	Exp.	Theor.	Exp.	Theor.
A	1640.63^1,2^	*1639*.*839 * ^1,2^	820.82^1,2^	*820*.*423 * ^1,2^	547.55^1,2^	*547*.*282 * ^1,2^	TWEECCKNPGCR
B	1056.39^1,2^	*1055.180* ^1,2^	528.70^1,2^	*528.094* ^1,2^	—	x	TWEECCK
C	754.37	*754*.*359 *	377.69	*377.896 *	—	*(252.125) *	NNHVDR
D	619.30^2^	*619*.*298 * ^2^	—^3^	*(310.366)* ^2,4^	—	x	CRGQV
E	603.28^2^	*603*.*267 * ^2^	—	*(302.137) *	—	x	NPGCR
F	335.16^2^	*335*.*147 * ^2^	—	x^5^	—	x	CR

^1^Acetylation (N-terminal, variable modification).

^2^Carbamidomethyl cysteine (fixed modification).

^3^Not detected.

^4^Not observed.

^5^Not expected.
