# Supplementary material for: α-RgIB: A Novel Antagonist Peptide of Neuronal Acetylcholine Receptor Isolated from Conus regius Venom
Source: Int J Pept. 2013 Feb 27;2013:543028. doi: 10.1155/2013/543028 (PMC3600190; doi:10.1155/2013/543028)

**α-RgIB: a novel antagonist peptide of neuronal acetylcholine receptor isolated from *Conus regius* venom – Supplementary material**

Maria Cristina Vianna Braga^1,§^, Arthur Andrade Nery^2^, Henning Ulrich^2^, Katsuhiro Konno^3^, Juliana Mozer Sciani^4^, Daniel Carvalho Pimenta^4,*^

^1^CAT/CEPID, Instituto Butantan. Avenida Vital Brasil, 1500. São Paulo, SP, 05503-900, Brazil.

^2^Departamanto de Bioquímica, IQ/USP. P.O. Box: 26077- Sao Paulo, SP, 05508-900, Brazil

^3^Institute of Natural Medicine, University of Toyama. 2630 Sugitani, Toyama 930-0194, Japan

^4^Laboratório de Bioquímica e Biofísica, Instituto Butantan. Avenida Vital Brasil, 1500. São Paulo, SP, 05503-900, Brazil.

* Corresponding author:

Avenida Vital Brazil, 1500.

São Paulo - SP

05503-900

Brazil

Phone: + 55 11 3726-7222 ext 2101

Fax: + 55 11 3726-7222 ext 2018

e-mail: [dcpimenta@butantan.gov.br](mailto:dcpimenta@butantan.gov.br)

^§^Present address:

Ministério da Ciência e Tecnologia

Esplanada dos Ministérios, Bloco E
Brasília, DF

70067-900

Brazil

**Supplementary figure 1: ClustalW alignment of UniProt-deposited α-conotoxins**

Rg1b ----TWEECCK---NPGCRNNHVD-----RCRGQV---------------

Q9XZK7 -------ECCS---HPACNVDHPE-----ICR------------------

Q9XZK6 -------ECCS---DPRCNSSHPE-----LCG------------------

Q9U619 ------DYCCH---RGPCMVWC----------------------------

Q9BP56 -------GCCS---RPPCALSNPD-----YC-------------------

Q8I6R5 -------ACCS---DRRCRWRC----------------------------

Q86RB2 -------GCCS---HPACAGNNQH-----IC-------------------

Q6PTD7 ------DECCP---DPPCKASNPD-----LCDWRS---------------

Q6PTD6 -------QCCA---NPPCKHVN--------CR------------------

Q6PTD5 -------GCCA---RAACAGIHQE-----LCG------------------

Q6PTD4 -------GCCS---HPACSVNNPY-----FCG------------------

Q6PTD1 -------GCCS---HPACSVNNPD-----IC-------------------

Q6PPB3 ------NECCD---NPPCKSSNPD-----LCDWRS---------------

Q2I2R8 -------GCCA---RAACAGIHQE-----LCG------------------

Q2I2R7 -------GCCA---RAACAGIHQE-----LCG------------------

Q2I2R6 -------ECCD---DPPCRQNNME-----HCPAS----------------

Q1L777 -------GCCS---HPACSVNHPE-----LC-------------------

P86261 -------GCCS---RPPCIANNPD-----IC-------------------

P85886 ------RTCCS---RPTCRMEYPE-----LCG------------------

P85013 -------GCCS---HPACNVNNPH-----IC-------------------

P85012 ------DYCCR---RPPCTLIC----------------------------

P85011 -------GCCS---DPRCRYRC----------------------------

P85010 -------GCCS---DPRCKHEC----------------------------

P85009 -------GCCS---DPRCKHQC----------------------------

P69747 -------GCCS---DPRCNYDHPE-----IC-------------------

P69746 --------CCG-VPNAACHPCVCK----NTC-------------------

P69658 -----RDPCCS---NPVCTVHNPQ-----IC-------------------

P69657 -------GCCS---TPPCAV---L-----YC-------------------

P60274 ----IRDECCS---NPACRVNNPH-----VC-------------------

P58783 -------GCCGKYPNAACHPCGCTVGRPPYCDRPSGG-------------

P58782 -------GCCGPYPNAACHPCGCKVGRPPYCDRPSGG-------------

P56973 --------CC----HPACGKYYS-------C-------------------

P56641 -------GCCS---YPPCFATNSG-----YC-------------------

P56640 -------GCCS---YPPCFATNPD------C-------------------

P56639 -------GCCS---YPPCFATNSD-----YC-------------------

P56638 -------GCCS---DPRCNMNNPD-----YC-------------------

P56636 -------GCCS---NPVCHLEHSN-----LC-------------------

P55963 -------GCCGSYPNAACHPCSCK-DRPSYCGQ-----------------

P50985 -------GCCS---LPPCALSNPD-----YC-------------------

P50984 -------GCCS---LPPCAANNPD-----YC-------------------

P50983 -------GCCS---DPRCAWRC----------------------------

P50982 -----RDPCCY---HPTCNMSNPQ-----IC-------------------

P28879 ------GCCC----NPACGPNYG-------CGTSCS--------------

P28878 -------YCC----HPACGKNFD-------C-------------------

P15471 -------ICC----NPACGPKYS-------C-------------------

P0CI05 ------GGCCS---HPACQNNPDY------C-------------------

P0CE74 -------ECCT---HPACHVSHPE-----LC-------------------

P0CE73 -------GCCS---HPACSVNNPD-----IC-------------------

P0CB13 ------PECCS---DPRCNSTHPE-----LCG------------------

P0CB12 ------QECCS---YPACNLDHPE-----LC-------------------

P0CB08 ------PECCS---DPRCNSTHPE-----LCG------------------

P0CB07 ------QECCS---YPACNLDHPE-----LC-------------------

P0CAQ9 ------DGCCP---NPSCSVNNPD-----IC-------------------

P0CAQ8 ------DGCCS---NPSCSVNNPD-----IC-------------------

P0CAQ5 -----NGRCC----HPACGKHFS-------C-------------------

P0CAQ4 -----NGRCC----HPACGKHFN-------C-------------------

P0C901 -------SCCS---DSDCNANHPD-----MCS------------------

P0C8V4 ------GGCCS---YPPCAVSNPQ-----HC-------------------

P0C8V1 ----GRGRCC----HPACGPNYS-------C-------------------

P0C8V0 ISEMTWEECCT---NPVCRQHYMH-----YC-------------------

P0C8U9 NAWLTPEECCA---APACREMILE-----FCLAGEAFAAALDGFRRLPYR

P0C8U8 NAWFTPEECCA---APACRGMILE-----FCLAGEAFAAALDGFRRLPYR

P0C8U7 ------DDCCP---DPACRQNHPE-----ICPSR----------------

P0C8U6 ------DDCCP---DPACRQNHPE-----LCSTR----------------

P0C8U5 ------GRCC----HPACAKYFS-------C-------------------

P0C8U4 -----NGRCC----HPACGKHFS-------C-------------------

P0C8U3 ---IT---CCT---RGTCAQH---------C-------------------

P0C8R2 ------DECCS---NPACRVNNPH-----VC-------------------

P0C351 ----QSPGCCW---NPACVKNRC---------------------------

P0C1Y2 ------DYCCH---RGPCMVWC----------------------------

P0C1X0 --------CCG-IPNAACHPCVCT----GKC-------------------

P0C1W9 --------CCG-VPNAACHPCVCT----GKC-------------------

P0C1W8 --------CCG-VPNAACPPCVCN----KTCG------------------

P0C1W4 -------GCCR---NPACESHRC---------------------------

P0C1W2 -----NGRCC----HPACARKYN-------C-------------------

P0C1W1 -----DGRCC----HPACAKHFN-------C-------------------

P0C1W0 -------GCCA---IRECRLQNAA-----YC-------------------

P0C1V9 ---QN---CCS---IPSCWEKYKC-----S--------------------

P0C1V8 ------GGCCS---HPACFASNPD-----YC-------------------

P0C1V7 --------CCS---HPACAANNQD-----YC-------------------

P0C1R7 -------GCCS---HPACNVNNPH-----ICG------------------

P0C1D0 -------GCCS---DPRCRYRC----------------------------

P01521 ------GRCC----HPACGKNYS-------C-------------------

P01520 -------ECC----HPACGKHFS-------C-------------------

P01519 -------ECC----NPACGRHYS-------C-------------------

A6M938 ------NDCCH---NAPCRNNHPG-----IC-------------------

A6M934 -------GCCS---NPPCYANNQA-----YCN------------------

A1X8D9 ------LSCCA---DPACKHTPG-------C-------------------

A1X8D8 ------GGCCS---YPPCIANNPL------CG------------------

A1X8D6 --------CCS---DSDCNANHPD-----MCS------------------

A1X8D1 -------GCCS---NPTCAGNNGN-----IC-------------------

A1X8D0 -------GCCS---NPACSVNHPE-----LC-------------------

A1X8C6 ------DDCCP---NPPCKASNPD-----LCDWRS---------------

A1X8C3 ---GMWDECCD---DPPCRQNNME-----HCPAS----------------

A1X8C2 ---GVWDECCK---DPQCRQNHMQ-----HCPAR----------------

A1X8B6 -------GCCS---HPACSGNHQE-----LCD------------------

A0SE60 ---QN---CCN---VPGCWAKYKH-----LC-------------------

A0SE59 -------GCCS---HPACRVHYPH-----VCY------------------

**Supplementary Table 1: Accession number, toxin name and species of origin of the analyzed conotoxins**

| Accession | Protein names | Organism |
| --- | --- | --- |
| Q9XZK7 | Alpha-conotoxin-like Tx2 | Conus textile (Cloth-of-gold cone) |
| Q9XZK6 | Alpha-conotoxin-like Tx1 | Conus textile (Cloth-of-gold cone) |
| Q9U619 | Alpha-conotoxin-like ImIIA | Conus imperialis (Imperial cone) |
| Q9BP56 | Alpha-conotoxin-like PnMGMR-02 | Conus pennaceus (Feathered cone) (Conus episcopus) |
| Q8I6R5 | Alpha-conotoxin ImII (Alpha-CTx ImII) (Fragment) | Conus imperialis (Imperial cone) |
| Q86RB2 | Alpha-conotoxin GIC (Fragment) | Conus geographus (Geography cone) (Nubecula geographus) |
| Q6PTD7 | Alpha-conotoxin-like Qc1.1a | Conus quercinus (Oak cone) |
| Q6PTD6 | Alpha-conotoxin-like Qc1.2 | Conus quercinus (Oak cone) |
| Q6PTD5 | Alpha-conotoxin Lp1.1 | Conus leopardus (Leopard cone) |
| Q6PTD4 | Alpha-conotoxin-like Lp1.2 | Conus leopardus (Leopard cone) |
| Q6PTD1 | Alpha-conotoxin-like Mr1.1 | Conus marmoreus (Marble cone) |
| Q6PPB3 | Alpha-conotoxin-like Qc1.1b (Qc1.3) | Conus quercinus (Oak cone) |
| Q6PPB2 | Alpha conotoxin QC1.4 | Conus quercinus (Oak cone) |
| Q2I2R8 | Alpha-conotoxin-like Lt1.1 (Lt1a) | Conus litteratus (Lettered cone) |
| Q2I2R7 | Alpha-conotoxin-like Lt1.2 (Lt1b) | Conus litteratus (Lettered cone) |
| Q2I2R6 | Alpha-conotoxin-like Lt1.3 (Lt1c) | Conus litteratus (Lettered cone) |
| Q1L777 | Alpha-conotoxin PeIA (Fragment) | Conus pergrandis (Grand cone) |
| P86261 | Alpha-conotoxin-like 1 | Conus textile (Cloth-of-gold cone) |
| P85886 | Alpha-conotoxin SrIA/SrIB | Conus spurius (Alphabet cone) |
| P85013 | Alpha-conotoxin-like Reg2a | Conus regius (Crown cone) |
| P85012 | Alpha-conotoxin-like Reg1f | Conus regius (Crown cone) |
| P85011 | Alpha-conotoxin-like Reg1e | Conus regius (Crown cone) |
| P85010 | Alpha-conotoxin-like Reg1d | Conus regius (Crown cone) |
| P85009 | Alpha-conotoxin-like Reg1b/Reg1c | Conus regius (Crown cone) |
| P69747 | Alpha-conotoxin Vc1A (ACV1) (Alpha-Vc1A) (Vc1.1) | Conus victoriae (Queen Victoria cone) |
| P69746 | Alpha-conotoxin OIVA (Alpha-A-O4a) (Alpha-A-OIVA) | Conus obscurus (Obscure cone) (Conus halitropus) |
| P69658 | Alpha-conotoxin PIA (Fragment) | Conus purpurascens (Purple cone) |
| P69657 | Alpha-conotoxin BuIA (Conotoxin Bu1.3) | Conus bullatus (Bubble cone) |
| P60274 | Alpha-conotoxin GID | Conus geographus (Geography cone) (Nubecula geographus) |
| P58783 | Alpha-conotoxin EIVB | Conus ermineus (Atlantic fish-hunting cone) |
| P58782 | Alpha-conotoxin EIVA | Conus ermineus (Atlantic fish-hunting cone) |
| P56973 | Alpha-conotoxin CnIA [Cleaved into: Alpha-conotoxin CnIB] | Conus consors (Singed cone) |
| P56641 | Alpha-conotoxin AuIC | Conus aulicus (Court cone) |
| P56640 | Alpha-conotoxin AuIB | Conus aulicus (Court cone) |
| P56639 | Alpha-conotoxin AuIA | Conus aulicus (Court cone) |
| P56638 | Alpha-conotoxin-like EpI | Conus episcopatus (Bishop's cone) |
| P56636 | Alpha-conotoxin MII (Alpha-MII) (CtxMII) (Alpha-conotoxin M2) | Conus magus (Magus cone) (Magician's cone snail) |
| P55963 | Alpha-conotoxin PIVA | Conus purpurascens (Purple cone) |
| P50985 | Alpha-conotoxin PnIB | Conus pennaceus (Feathered cone) (Conus episcopus) |
| P50984 | Alpha-conotoxin PnIA (Alpha-PnIA) | Conus pennaceus (Feathered cone) (Conus episcopus) |
| P50983 | Alpha-conotoxin ImI (Alpha-CTx ImI) (Fragment) | Conus imperialis (Imperial cone) |
| P50982 | Alpha-conotoxin EI | Conus ermineus (Atlantic fish-hunting cone) |
| P28879 | Alpha-conotoxin S2 (Alpha-conotoxin SII) | Conus striatus (Striated cone) |
| P28878 | Alpha-conotoxin SIA (S1A) | Conus striatus (Striated cone) |
| P15471 | Alpha-conotoxin S1 (SI) | Conus striatus (Striated cone) |
| P0CI05 | Alpha-conotoxin | Conus tinianus (Variable cone) |
| P0CE74 | Alpha-conotoxin-like Bn1.2 (Fragment) | Conus bandanus (Banded marbled cone) |
| P0CE73 | Alpha-conotoxin-like Bn1.1 | Conus bandanus (Banded marbled cone) |
| P0CB13 | Alpha-conotoxin-like 291 | Conus ammiralis (Admiral cone) |
| P0CB12 | Alpha-conotoxin-like 289 | Conus ammiralis (Admiral cone) |
| P0CB08 | Alpha-conotoxin-like Ai1.2 | Conus ammiralis (Admiral cone) |
| P0CB07 | Alpha-conotoxin-like Ai1.1 | Conus ammiralis (Admiral cone) |
| P0CAQ9 | Alpha-conotoxin-like Qc1.4b (Fragment) | Conus quercinus (Oak cone) |
| P0CAQ8 | Alpha-conotoxin-like Qc1.4a (Fragment) | Conus quercinus (Oak cone) |
| P0CAQ5 | Alpha-conotoxin-like Ac1.1b | Conus achatinus (Little frog cone) |
| P0CAQ4 | Alpha-conotoxin-like Ac1.1a | Conus achatinus (Little frog cone) |
| P0C901 | Alpha-conotoxin-like Leo-A1 | Conus leopardus (Leopard cone) |
| P0C8V4 | Alpha-conotoxin-like Vn | Conus ventricosus (Mediterranean cone) |
| P0C8V1 | Alpha-conotoxin-like Sm1.1 | Conus stercusmuscarum (Fly-specked cone) |
| P0C8V0 | Alpha-conotoxin-like Pu1.6 | Conus pulicarius (Flea-bite cone) |
| P0C8U9 | Alpha-conotoxin-like Pu1.5 | Conus pulicarius (Flea-bite cone) |
| P0C8U8 | Alpha-conotoxin-like Pu1.4 | Conus pulicarius (Flea-bite cone) |
| P0C8U7 | Alpha-conotoxin-like PuSG1.2 | Conus pulicarius (Flea-bite cone) |
| P0C8U6 | Alpha-conotoxin-like PuSG1.1 | Conus pulicarius (Flea-bite cone) |
| P0C8U5 | Alpha-conotoxin-like Mn1.4 (Fragment) | Conus monachus (Cone snail) |
| P0C8U4 | Alpha-conotoxin-like Cn1.1 | Conus consors (Singed cone) |
| P0C8U3 | Alpha-conotoxin-like Br1.4 | Conus brunneus (Wood's brown cone) |
| P0C8R2 | Alpha-conotoxin ArIA [Cleaved into: Alpha-conotoxin ArIB] | Conus arenatus (Sand-dusted cone) |
| P0C351 | Alpha-conotoxin PIB | Conus purpurascens (Purple cone) |
| P0C1Y2 | Alpha-conotoxin-like Bn1.3 | Conus bandanus (Banded marbled cone) |
| P0C1X0 | Alpha-conotoxin PeIVB | Conus pergrandis (Grand cone) |
| P0C1W9 | Alpha-conotoxin PeIVA | Conus pergrandis (Grand cone) |
| P0C1W8 | Alpha-conotoxin OIVB (Alpha-A-O4b) (Alpha-A-OIVB) | Conus obscurus (Obscure cone) (Conus halitropus) |
| P0C1W4 | Alpha-conotoxin-like S1.1 | Conus striatus (Striated cone) |
| P0C1W2 | Alpha-conotoxin-like MIB | Conus magus (Magus cone) (Magician's cone snail) |
| P0C1W1 | Alpha-conotoxin-like MIA | Conus magus (Magus cone) (Magician's cone snail) |
| P0C1W0 | Alpha-conotoxin-like Ca1.2 | Conus caracteristicus (Characteristic cone) |
| P0C1V9 | Alpha-conotoxin-like Ca1.1 | Conus caracteristicus (Characteristic cone) |
| P0C1V8 | Alpha-conotoxin-like AnIC | Conus anemone (Anemone cone) |
| P0C1V7 | Alpha-conotoxin AnIB [Cleaved into: Alpha-conotoxin AnIA] | Conus anemone (Anemone cone) |
| P0C1R7 | Alpha-conotoxin OmIA | Conus omaria (Omaria cone) |
| P0C1D0 | Alpha-conotoxin RgIA (Fragment) | Conus regius (Crown cone) |
| P01521 | Alpha-conotoxin MI (Alpha-MI) (CtxMI) (M1) | Conus magus (Magus cone) (Magician's cone snail) |
| P01520 | Alpha-conotoxin GII | Conus geographus (Geography cone) (Nubecula geographus) |
| P01519 | Alpha-conotoxin GIA [Cleaved into: Alpha-conotoxin GI (G1)] | Conus geographus (Geography cone) (Nubecula geographus) |
| A6M938 | Alpha-conotoxin-like Lp1.10 (Fragment) | Conus leopardus (Leopard cone) |
| A6M934 | Alpha-conotoxin-like Mr1.2 | Conus marmoreus (Marble cone) |
| A1X8D9 | Alpha-conotoxin-like Pu1.3 (Fragment) | Conus pulicarius (Flea-bite cone) |
| A1X8D8 | Alpha-conotoxin-like Pu1.2 (Fragment) | Conus pulicarius (Flea-bite cone) |
| A1X8D6 | Alpha-conotoxin-like Lp1.9 (Fragment) | Conus leopardus (Leopard cone) |
| A1X8D1 | Alpha-conotoxin-like Qc1.6 (Fragment) | Conus quercinus (Oak cone) |
| A1X8D0 | Alpha-conotoxin-like Qc1.5 (Fragment) | Conus quercinus (Oak cone) |
| A1X8C6 | Alpha-conotoxin-like Qc1.1c (Fragment) | Conus quercinus (Oak cone) |
| A1X8C3 | Alpha-conotoxin-like Lp1.7 (Alpha-conotoxin-like Lp1.8) | Conus leopardus (Leopard cone) |
| A1X8C2 | Alpha-conotoxin-like Lp1.8 (Alpha-conotoxin-like Lp1.7) | Conus leopardus (Leopard cone) |
| A1X8B6 | Alpha-conotoxin-like Lp1.4 | Conus leopardus (Leopard cone) |
| A0SE60 | Alpha-conotoxin-like Pu1.1 | Conus pulicarius (Flea-bite cone) |
| A0SE59 | Alpha-conotoxin-like Mr1.3 | Conus marmoreus (Marble cone) |

**Supplementary figure 2: Figure. Molecular Phylogenetic anaylsis of α-conotoxins by Maximum Likelihood method** .

Black diamond: α-rgIb; white triangle: α-RgIA

The evolutionary history was inferred by using the Maximum Likelihood method based on the JTT matrix-based model [1]. The tree with the highest log likelihood (-2079.0738) is shown. Initial tree(s) for the heuristic search were obtained automatically as follows. When the number of common sites was < 100 or less than one fourth of the total number of sites, the maximum parsimony method was used; otherwise BIONJ method with MCL distance matrix was used. The tree is drawn to scale, with branch lengths measured in the number of substitutions per site. The analysis involved 105 amino acid sequences. All ambiguous positions were removed for each sequence pair. There were a total of 53 positions in the final dataset. Evolutionary analyses were conducted in MEGA5 [2].

1. Jones D.T., Taylor W.R., and Thornton J.M. (**1992**). The rapid generation of mutation data matrices from protein sequences. *Computer Applications in the Biosciences* **8**: 275-282.

2. Tamura K., Peterson D., Peterson N., Stecher G., Nei M., and Kumar S. (**2011**). MEGA5: Molecular Evolutionary Genetics Analysis using Maximum Likelihood, Evolutionary Distance, and Maximum Parsimony Methods. *Molecular Biology and Evolution* **(In Press)**.

**Supplementary figure 3: Expression profile of the neuronal nicotine receptors on the fifth day of the PC12 cell culture, as revealed by a 35-cycle PCR**

1018

505

bp

**α2 α3 α4 α5 α7 β2 β3 β4**

**⮦ β-actine**

1353

603

217

bp


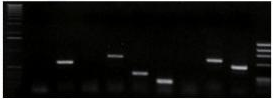

Supplement: Supplementary file 1 — The accompanying figures and tables provide information that subside the results presented in the main text. These data consist of one CLUSTALW alignment of all deposited α-conotoxins, one explanatory table with the toxin description and organism of origin and the resulting molecular phylogenetic tree built (by MEGA) on the alignment data. This tree branches provide a better visualization of the possible phylogenetic relations among the toxins. This tree served to locate the branch in which α-RgIB sits. The closest phylogenetic relatives were than reanalyzed for homology and presented in the main text. Moreover, this material also presents the expression profile of the nicotinic receptors detected in the employed cell cultures, which is important for proper pharmacological characterization. [file 543028.f1.docx]
